# Elevated blood urea nitrogen-to-creatinine ratio predicts short-term mortality in intensive care unit patients with ischemic stroke: Evidence from a multicenter cohort

**DOI:** 10.1371/journal.pone.0337807

**Published:** 2025-12-04

**Authors:** Huang Luwen, Wu Dongmei, Yu Ming, Xu Lei, Zhang Yunwei

**Affiliations:** Department of Neurology, Suining Central Hospital, Suining, Sichuan, China; University of Health Sciences, Beyhekim Training and Research Hospital, TÜRKIYE

## Abstract

**Background:**

The blood urea nitrogen-to-creatinine ratio (BUCR) has emerged as a potential biomarker for predicting the prognosis of Intensive Care Unit (ICU) patients. However, its relationship with short-term mortality in patients with ischemic stroke (IS) remains controversial. This study aimed to investigate the association between BUCR and 28-day in-hospital mortality in ICU IS patients.

**Methods:**

This retrospective cohort study utilized data from the eICU Collaborative Research Database (2014--2015). A total of 2,702 ICU patients with IS were included. The BUCR was calculated on the basis of initial blood urea nitrogen and serum creatinine levels obtained within the first 24 hours of ICU admission. The association between BUCR and 28-day in-hospital mortality was assessed via Cox proportional hazards regression models. Restricted cubic spline analysis was performed to evaluate potential nonlinear relationships. Sensitivity analyses—including complete-case analysis, multiple imputation, propensity score matching, and E-value estimation—were conducted to assess the robustness of the findings.

**Results:**

The mean age at baseline was 68.59 years (SD: 14.29), and 313 patients (11.58%) died within 28 days of hospitalization. Compared with Q1, BUCR in Q3 was significantly associated with 28-day in-hospital mortality (HR = 1.616; 95% CI: 1.173, 2.226). After full adjustment for potential confounders, a linear relationship was observed between BUCR and mortality risk. Each unit increase in BUCR was associated with a 1.4% increase in the hazard of death (HR = 1.014; 95% CI: 1.002, 1.027; P = 0.021). Sensitivity analyses confirmed the robustness of this association.

**Conclusion:**

An elevated BUCR is an independent predictor of 28-day in-hospital mortality in ICU patients with ischemic stroke. This finding provides compelling evidence to address existing inconsistencies in the field, suggesting that BUCR may serve as a simple and effective biomarker for identifying high-risk patients with severe IS.

## Introduction

Stroke remains a leading cause of death worldwide, with ischemic stroke (IS) being the most prevalent subtype [1–3]. Intensive Care Unit (ICU) patients with IS are at particularly high risk of short-term mortality [4], underscoring the importance of early and reliable prognostic markers to support clinical decision-making. Numerous factors have been associated with IS-related mortality, including malnutrition [5], low-density lipoprotein cholesterol [6], hemoglobin glycation [7], hypertension [8], and diabetes mellitus (DM) [9]. However, additional predictive indicators are needed to improve risk stratification and guide therapeutic strategies in high-risk patients.

The blood urea nitrogen-to-creatinine ratio (BUCR) has traditionally served as a biomarker for renal function, as both components reflect end products of nitrogen metabolism. More recently, BUCR has been applied in the prognostic evaluation of various nonrenal conditions, including heart failure [10], insulin resistance [11], acute pancreatitis [12], and obstructive sleep apnea [13]. Several studies have also suggested that BUCR is an independent predictor of adverse outcomes in IS patients [14,15]. However, some studies have reported no significant association between BUCR and IS prognosis [16,17]. These discrepancies may be attributed to methodological differences, including single-center study designs, limited sample sizes, and inadequate control for confounding variables.

To address these inconsistencies, the present study utilized data from the multicenter eICU Collaborative Research Database to further investigate the relationship between BUCR and short-term mortality in ICU IS patients. This analysis aims to fill existing gaps in the literature and provide more robust evidence for the clinical application of BUCR as a prognostic biomarker.

## Methods

### Data source

We used data from the eICU Collaborative Research Database (version 2.0), which contains deidentified clinical data from over 200,000 ICU admissions across multiple centers in the United States between 2014 and 2015. The database includes comprehensive information such as demographics, diagnoses, laboratory measurements, vital signs, and clinical outcomes. Database access was approved following CITI certification and data use agreement authorization. One of the authors (Huang Luwen) obtained the necessary credentials to access the dataset. All analyses were conducted in accordance with the Health Insurance Portability and Accountability Act regulations and the principles of the Declaration of Helsinki.

### Study population

**Fig 1:**
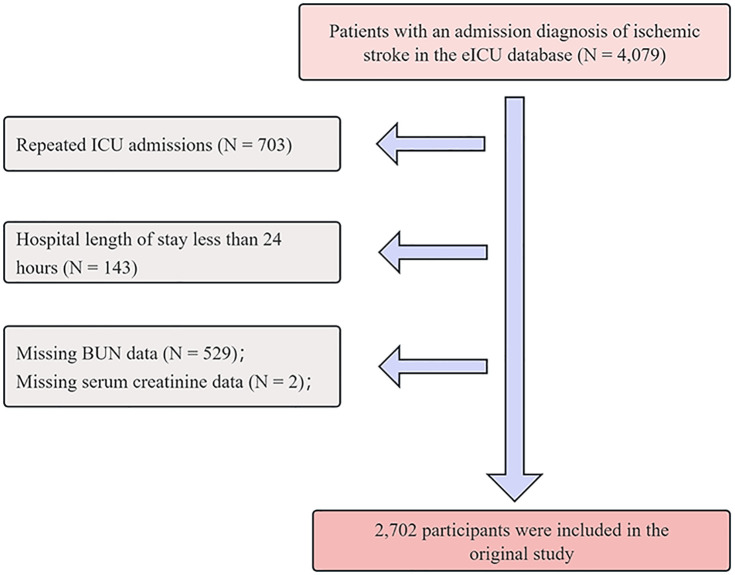
Flow chart of the study population.

A total of 4,079 patients diagnosed with IS were initially identified via the search terms “neurologic | disorders of vasculature | stroke | ischemic stroke” [18]. To enhance dataset quality and minimize bias, exclusions were made on the basis of the following criteria: (1) repeated ICU admissions (n = 703); (2) hospital stay less than 24 hours (n = 143); (3) missing blood urea nitrogen (BUN) data (n = 529); and (4) missing serum creatinine (Scr) data (n = 2). After applying these criteria, 2,702 patients were included in the final analysis. Fig 1 displays the flow of patient selection.

### Exposure and Outcome Definitions

The exposure variables BUCR were calculated via the following standard formula:

BUCR = BUN (mg/dL)/Scr (mg/dL) [13,19];

The primary outcome was 28-day all-cause in-hospital mortality.

### Assessments of covariates

The extracted variables included demographic characteristics (sex, age, ethnicity, and body mass index), clinical indicators (mechanical ventilation use and sequential organ failure assessment [SOFA] score), comorbid conditions (sepsis, chronic obstructive pulmonary disease, congestive heart failure [CHF], acute myocardial infarction, DM, pneumonia, and arrhythmia), and laboratory parameters, including glucose, BUN, Scr, serum potassium, and serum sodium levels. Additionally, diagnostic information was extracted from the eICU database’s diagnosis table, which records active diagnoses during the ICU stay. Each diagnosis included a diagnostic string (e.g., “pulmonary disorders of the airways”) and the corresponding ICD-9 code (e.g., 518.81). The diagnosis priority (diagnosisPriority) indicated whether the diagnosis was primary, major, or other, and although this field was not mandatory, it was used to assess the severity of the diagnosis and its relevance to the patient’s condition.

### Missing variables

The distribution of missing data is summarized in S1 Table. Multiple imputation (MI) via chained equations was conducted under the assumption that data were missing at random, generating five datasets that were pooled using Rubin’s rules to account for imputation variability. In addition, a complete-case analysis was performed as a sensitivity analysis. Together, these approaches preserve analytical integrity and allow for assessment of the robustness of the results.

### Statistical analysis

Continuous variables are summarized as the means ± SDs or medians (IQRs), and categorical variables are summarized as frequencies (percentages).

To assess potential collinearity between the BUCR and other covariates, we evaluated the generalized variance inflation factor (GVIF) and adjusted GVIF (S2 Table). Both univariate and multivariate Cox proportional hazards regression was used to estimate hazard ratios (HRs) for 28-day mortality. Model 1 was unadjusted. Model 2 was adjusted for age, sex, and ethnicity. Model 3 further included mechanical ventilation, the SOFA score, DM, sepsis, chronic obstructive pulmonary disease, CHF, acute myocardial infarction, arrhythmia, pneumonia, and serum levels of potassium and sodium.

Cumulative incidence curves based on a competing risk model were generated to depict 28-day in-hospital mortality across different BUCR categories. Restricted cubic spline (RCS) analysis was conducted to assess the potential dose–response association between BUCR and 28-day mortality. Receiver operating characteristic (ROC) curves were generated to evaluate and compare the predictive accuracy of BUN, Scr, and BUCR levels. The area under the ROC curve (AUC) was used to assess the discriminative capacity of each variable. Subgroup analyses were conducted via multivariate Cox regression models across predefined categories, including age, sex, ethnicity, DM, arrhythmia, pneumonia, and laboratory parameters.

To further assess the robustness of our findings, several sensitivity analyses were performed. First, Cox regression was repeated using a complete-case dataset, excluding all observations with missing values. Second, the analysis was repeated using the MIdataset to account for missing data. Third, a propensity score weighted analysis was performed to assess the association between BUCR and 28-day in-hospital mortality. BUCR was categorized into three groups: Q1, Q2, and Q3. Multiple weighting methods, including inverse probability of treatment weighting (IPTW), overlap weighting (OW), matching weighting, entropy weighting, and treated weighting, were applied to adjust for potential confounding factors. Fourth, an E value analysis was conducted to estimate the minimum strength of association that an unmeasured confounder would need to have with both BUCR and the outcome to fully explain the observed effect.

All the statistical analyses were performed via R software (version 3.3.2, The R Foundation) and Free Statistics software (version 1.7). A two-sided P value < 0.05 was considered statistically significant.

## Results

### Baseline characteristics

In this cohort of 2,702 ICU patients with IS, participants were stratified into three groups according to their BUCR: Q1 (<15), Q2 (15–20), and Q3 (>20) (Table 1). An increasing trend in age was observed across the BUCR categories (63.52 ± 14.42 vs. 68.70 ± 14.15 vs. 73.48 ± 12.48 years; P < 0.001), along with a progressively greater proportion of male patients (38.56% vs. 42.54% vs. 62.35%; P < 0.001). The high BUCR group presented elevated SOFA scores and a greater frequency of mechanical ventilation use (22.71%; P = 0.008). The prevalence of COPD, CHF, and arrhythmia was significantly greater in the high BUCR group than in the other groups (all P < 0.001). The laboratory parameters revealed a stepwise increase in blood glucose and BUN levels and a decrease in serum creatinine (Scr) concentrations across BUCR tertiles (all P < 0.001). The 28-day in-hospital mortality rate increased with increasing BUCR (8.79% vs. 10.64% vs. 15.28%; P < 0.001). When stratified by survival status, non-survivors had significantly greater BUCR values than survivors did (21.15 ± 10.14 vs. 18.76 ± 8.22; P < 0.001), as did survivors with higher SOFA scores, elevated BUN and Scr levels, and a markedly higher proportion of mechanical ventilation use (52.09% vs. 16.82%; all P < 0.001) (S3 Table). Univariate Cox regression analysis revealed that age ≥ 60 years, mechanical ventilation, SOFA score, arrhythmia, and elevated BUN were significantly associated with 28-day in-hospital mortality (S4 Table).

**Table 1:** Baseline characteristics of participants classified by the blood urea nitrogen to creatinine ratio.

Variables	Total	Low BUCR (< 15)	Middle BUCR (15-20)	High BUCR (>20)	P value
**N**	2702	887	912	903	
**Age, mean(SD), year**	68.59 ± 14.29	63.52 ± 14.42	68.70 ± 14.15	73.48 ± 12.48	< 0.001
**Gender(male), n(%)**	1293 (47.85)	342 (38.56)	388 (42.54)	563 (62.35)	< 0.001
**Ethnicity, n(%)**					< 0.001
** Caucasian**	2062 (80.99)	611 (72.48)	727 (85.33)	724 (85.08)	
** African American**	293 (11.51)	175 (20.76)	65 (7.63)	53 (6.23)	
** Other or unknown**	191 (7.50)	57 (6.76)	60 (7.04)	74 (8.7)	
**BMI, mean(SD), kg/m** ^**2**^	28.72 ± 7.19	29.15 ± 7.19	28.78 ± 6.82	28.24 ± 7.52	0.03
**Mechanical ventilation use, n(%)**	558 (20.94)	198 (22.68)	157 (17.48)	203 (22.71)	0.008
**SOFA score, Median (IQR)**	2.00 (1.00, 4.00)	2.00 (1.00, 4.00)	2.00 (1.00, 3.00)	2.00 (1.00, 4.00)	< 0.001
**SEPSIS, n(%)**	76 (2.81)	19 (2.14)	26 (2.85)	31 (3.43)	0.255
**COPD, n(%)**	127 (4.70)	32 (3.61)	31 (3.4)	64 (7.09)	< 0.001
**CHF, n(%)**	147 (5.44)	40 (4.51)	35 (3.84)	72 (7.97)	< 0.001
**AMI, n(%)**	82 (3.03)	24 (2.71)	25 (2.74)	33 (3.65)	0.412
**DM, n(%)**	559 (20.98)	166 (19.01)	195 (21.71)	198 (22.15)	0.216
**Pneumonia, n(%)**	210 (7.77)	58 (6.54)	68 (7.46)	84 (9.3)	0.084
**Arrhythmia, n (%)**	548 (20.28)	147 (16.57)	162 (17.76)	239 (26.47)	< 0.001
**Glucose, mean(SD), mg/dL**	138.52 ± 65.21	130.73 ± 55.27	140.82 ± 74.51	143.87 ± 63.50	< 0.001
**BUN, median(IQR), mg/dL**	17.00 (12.00, 24.00)	12.00 (9.00, 17.00)	16.00 (13.00, 21.00)	22.00 (17.00, 31.00)	< 0.001
**Serum creatinine, median(IQR), mg/dl**	0.91 (0.74, 1.21)	1.00 (0.80, 1.38)	0.92 (0.76, 1.20)	0.86 (0.67, 1.12)	< 0.001
**Serum potassium, mean(SD), mmol/L**	3.97 ± 0.57	3.94 ± 0.57	3.94 ± 0.53	4.02 ± 0.60	0.002
**Serum sodium, mean(SD), mmol/L**	139.07 ± 4.11	138.74 ± 3.85	138.88 ± 3.92	139.58 ± 4.48	< 0.001
**Hospital 28 mortality, n(%)**	313 (11.58)	78 (8.79)	97 (10.64)	138 (15.28)	< 0.001

BMI,body mass index; SOFA, sequential organ failure assessment; COPD, chronic obstructive pulmonary disease; CHF, congestive heart failure; AMI, acute myocardial infarction; DM, diabetes mellitus.

#### Associations of BUCR with 28-day in-hospital mortality.

Cox regression analysis revealed a significant positive association between BUCR and 28-day in-hospital mortality (Table 2). As a continuous variable, BUCR was independently associated with increased mortality across all the models: the HRs were 1.019 (95% CI: 1.008, 1.029) in Model 1, 1.013 (95% CI: 1.001, 1.025) in Model 2, and 1.014 (95% CI: 1.002, 1.027) in Model 3. When analyzed as tertiles and using Q1 as the reference, Q2 was significantly associated with higher mortality in Model 1 (HR = 1.402, P = 0.026) and Model 3 (HR = 1.586, P = 0.006), while a non-significant trend was shown in Model 2 (P = 0.079). All models showed statistically significant associations for Q3, with HRs ranging from 1.469 to 1.714 (all P < 0.05).

**Table 2:** Cox regression models for the association between the BUCR and 28-day in-hospital mortality.

Categories	Event, (n%)	Model 1		Model 2		Model 3	
		HR (95% CI)	P value	HR (95% CI)	P value	HR (95% CI)	P value
**BUCR index**							
**Continuous**	353 (10.9)	1.019 (1.008, 1.029)	< 0.001	1.013 (1.001, 1.025)	0.034	1.014 (1.002, 1.027)	0.021
**Groups**							
**Q1**	78 (8.8)	1(Ref)		1(Ref)		1(Ref)	
**Q2**	97 (10.6)	1.402 (1.041, 1.89)	0.026	1.324 (0.968, 1.813)	0.079	1.586 (1.14, 2.205)	0.006
**Q3**	138 (15.3)	1.714 (1.298, 2.263)	< 0.001	1.469 (1.083, 1.992)	0.013	1.616 (1.173, 2.226)	0.003
**P for trend**		1.301 (1.136, 1.49)	< 0.001	1.202 (1.036, 1.394)	0.015	1.244 (1.067, 1.451)	0.005

Model 1: unadjusted.

Model 2: adjusted for age, gender, and ethnicity.

Model 3: adjusted for Model 2 plus, BMI, mechanical ventilation use, SOFA score, DM, sepsis, COPD, CHF, AMI, arrhythmia, pneumonia, serum potassium, and serum sodium levels.

BMI, body mass index; SOFA, sequential organ failure assessment; COPD, chronic obstructive pulmonary disease; CHF, congestive heart failure; AMI, acute myocardial infarction; DM, diabetes mellitus.

**Fig 2:**
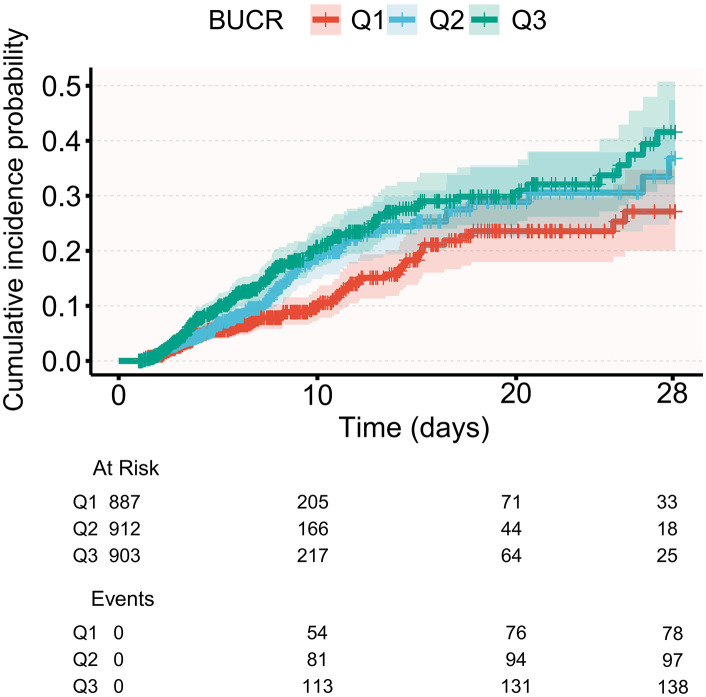
Cumulative incidence curves for 28-day in-hospital mortality by BUCR based on competing risk analysis. BUCR: blood urea nitrogen-to-creatinineratio.

**Fig 3:**
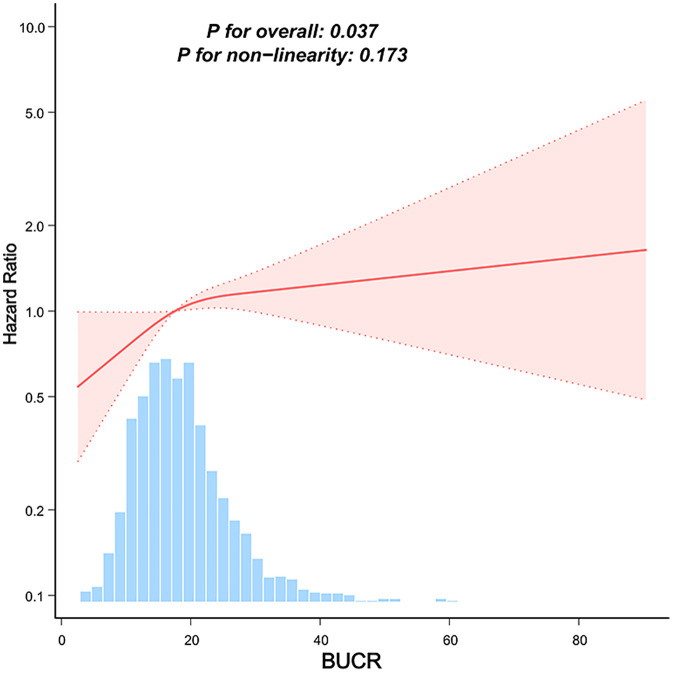
Restricted cubic spline models were used to analyze the relationship between BUCR and 28-day hospital mortality. **Adjusted** age, gender, ethnicity, BMI, mechanical ventilation use, SOFA score, DM, sepsis, COPD, CHF, AMI, arrhythmia, pneumonia, serum potassium, and serum sodium levels were adjusted for. BMI, body mass index; SOFA, sequential organ failure assessment; COPD, chronic obstructive pulmonary disease; CHF, congestive heart failure; AMI, acute myocardial infarction; DM, diabetes mellitus.

**Fig 4:**
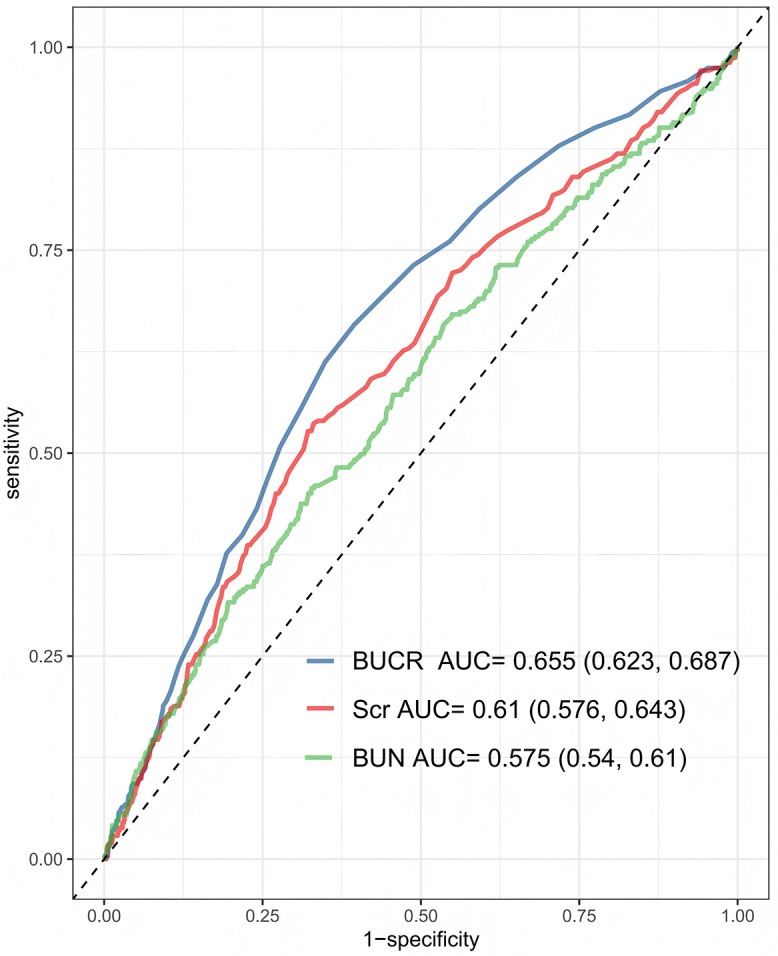
ROC curves comparing the predictive efficacy of the BUCR, Scr, and BUN for 28-day hospital mortality. BUCR, BUN/serum creatinine; Scr, serum creatinine; BUN, Blood Urea Nitrogen.

The cumulative incidence of mortality was estimated using a competing risk model (Fig 2). The resulting cumulative incidence curves showed clear and progressively widening separation across BUCR tertiles throughout the 28-day follow-up period, with the greatest divergence observed after day 10. By day 28, the number of mortality events was 131 in Q3, 94 in Q2, and 76 in Q1. Gray’s test confirmed a statistically significant difference in cumulative incidence among the three groups (P < 0.001), further supporting the association between elevated BUCR and increased short-term in-hospital mortality. BUCR (P for overall = 0.037) was significantly associated with an increased risk of mortality. with no evidence of nonlinear (P for nonlinear = 0.173), indicating an approximately linear relationship (Fig 3). Additionally, ROC analysis showed that BUCR had the strongest ability to predict 28-day in-hospital mortality among the three biomarkers (Fig 4). BUCR had the highest area under the curve (AUC = 0.655, 95% CI: 0.623, 0.687), followed by Scr (AUC = 0.61, 95% CI: 0.576, 0.643), while BUN demonstrated the lowest predictive performance (AUC = 0.575, 95% CI: 0.54, 0.61).

#### Subgroup analysis.

**Fig 5:**
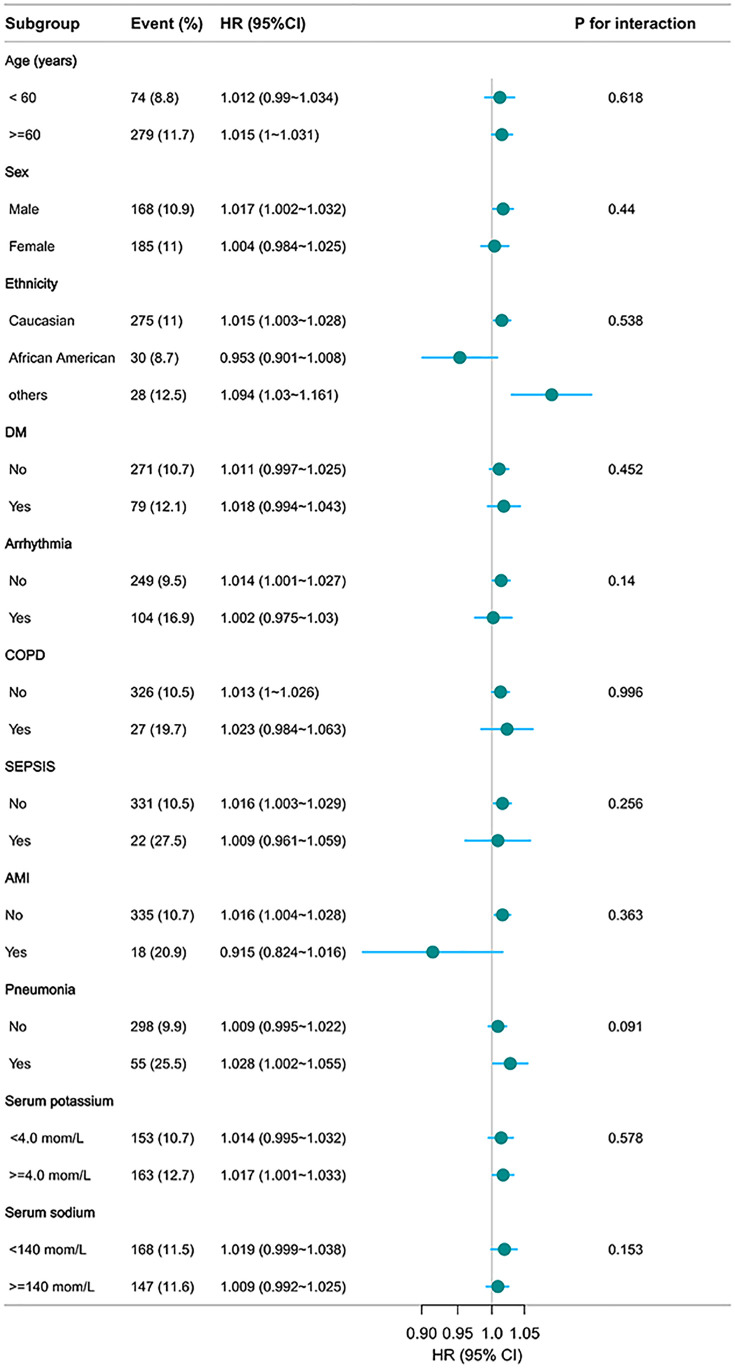
Subgroup analysis of BUCR in predicting 28-day in-hospital mortality. Age, gender, ethnicity, BMI, mechanical ventilation use, SOFA score, DM, sepsis, COPD, CHF, AMI, arrhythmia, pneumonia, serum potassium, and serum sodium levels were adjusted for. BMI, body mass index; SOFA, sequential organ failure assessment; COPD, chronic obstructive pulmonary disease; CHF, congestive heart failure; AMI, acute myocardial infarction; DM, diabetes mellitus.

Subgroup analysis demonstrated that the positive associations between BUCR and 28-day in-hospital mortality, including age, sex, ethnicity, comorbidities (DM, arrhythmia, COPD, sepsis, AMI, pneumonia), and serum potassium and sodium levels, remained consistent across all predefined strata (Fig 5). No significant interactions were detected (all P for interaction > 0.05), suggesting the absence of effect modification. The direction and magnitude of hazard ratios were comparable across subgroups, indicating that the association between elevated BUCR and short-term mortality was robust across clinical subpopulations.

#### Sensitivity analyses.

**Fig 6:**
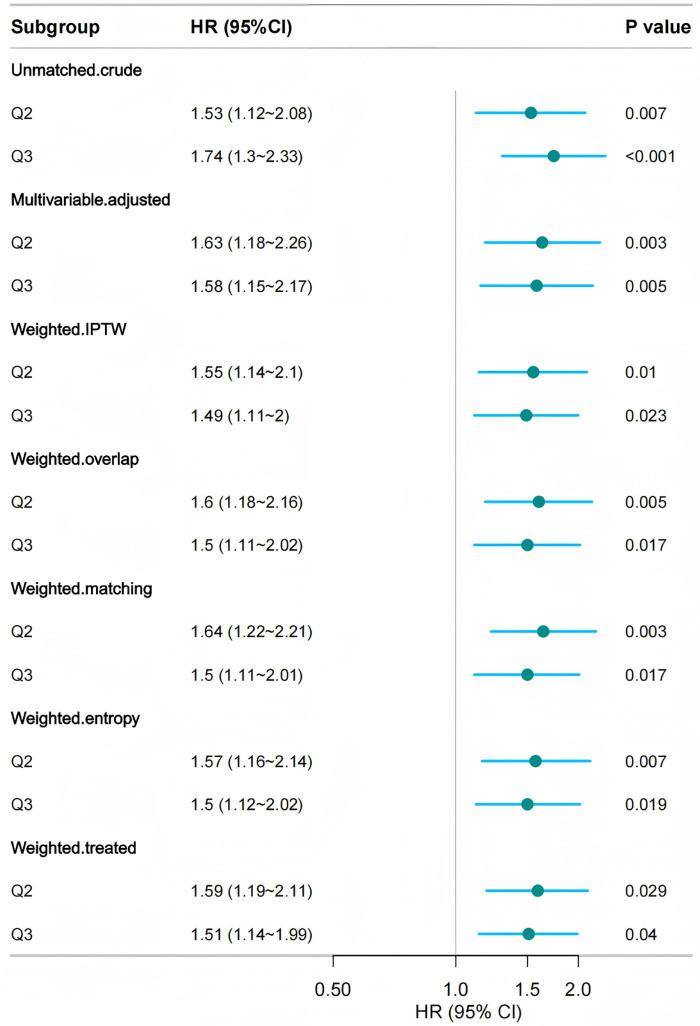
Association between BUCR and 28-day in-hospital mortality using multi-group propensity score weighting analysis. Adjusted age, gender, ethnicity, BMI, mechanical ventilation use, SOFA score, DM, sepsis, COPD, CHF, AMI, arrhythmia, pneumonia, serum potassium, and serum sodium levels were adjusted for. BMI, body mass index; SOFA, sequential organ failure assessment; COPD, chronic obstructive pulmonary disease; CHF, congestive heart failure; AMI, acute myocardial infarction; DM, diabetes mellitus.

First, in both the complete-case (S5 Table) and MI analyses (S6 Table), BUCR was positively associated with 28-day in-hospital mortality. According to the fully adjusted models, patients in Q2 had a higher risk of death than those in Q1 did (HRs = 1.586 and 1.466, both P < 0.01), with stronger associations observed in Q3 (HRs = 1.616 and 1.584, both P < 0.005). Similar results were obtained when BUCR was treated as a continuous variable. Second, in the propensity score weighted analysis, the three-group classification of BUCR demonstrated a significant association with 28-day in-hospital mortality (Fig 6). Regardless of the weighting method used (IPTW, overlap weighting, matching weighting, entropy weighting, or treated weighting), both the Q2 and Q3 groups were significantly associated with an increased risk of mortality. Specifically, the HR for Q2 ranged from 1.55 to 1.64 (P < 0.05), while the HR for Q3 ranged from 1.49 to 1.58 (P < 0.05). These findings indicate that higher BUCR values are significantly associated with an increased mortality risk. Third, E-value analysis revealed that a confounder would need a risk ratio of at least 2.61 with both BUCR and mortality to explain the observed association (S1 Fig). This makes residual confounding unlikely.

## Discussion

This study is the first to investigate the positive association between BUCR and 28-day in-hospital mortality among ICU patients with IS via the eICU database. After comprehensive adjustment for potential confounders, BUCR was independently associated with an increased risk of 28-day mortality. Furthermore, RCS analysis revealed a positive linear relationship between BUCR levels and mortality risk. These results remained robust across multiple sensitivity analyses, including complete-case analysis, MI, and various propensity score–based methods, confirming the stability of the association. These findings provide preliminary evidence supporting the potential utility of BUCR as a prognostic indicator in ICU patients with IS.

The BUCR, calculated as the ratio of BUN to Scr, is a simple yet clinically informative indicator derived from routine laboratory tests. Traditionally, it has been employed in nephrology and emergency care to evaluate renal perfusion, prerenal azotemia, and intravascular volume status [20]. More recently, its prognostic relevance has been explored in broader clinical settings. For example, several studies have reported that elevated BUCR is associated with poorer outcomes and increased mortality in patients with heart failure [21,22]. In the context of stroke, Anne et al. (2011) first proposed that BUCR could act as an indirect marker of dehydration in hospitalized stroke patients, with higher BUCR levels indicating greater dehydration severity and correlating with worse functional outcomes [23]. In support of this hypothesis, a prospective cohort study involving 324 AIS patients demonstrated that elevated BUCR values predict unfavorable 30-day prognoses [24]. Similarly, another study of 203 patients revealed that higher BUCR was associated with larger ischemic lesion volumes on diffusion-weighted imaging and more severe neurological deficits upon admission [14]. However, findings across studies have not been entirely consistent. For example, a large analysis of 3,059 AIS cases from the MIMIC-III and MIMIC-IV databases, including 2,085 PSM patients, revealed only a weak association between BUCR and in-hospital mortality (RR = 1.01) [15]. Furthermore, a separate investigation involving 3,355 AIS patients reported no significant associations between BUCR and either in-hospital mortality or poor discharge outcomes [16].

Our study, which was based on a large multicenter ICU cohort from the eICU Collaborative Research Database, revealed a robust, approximately linear association between BUCR and 28-day in-hospital mortality among IS patients. This finding extends current knowledge on the prognostic value of BUCR and underscores its relevance in a broader critical care context. In contrast, previous studies have reported nonlinear associations. Li et al., analyzing data from the MIMIC-IV database, reported a J-shaped association between BUCR and in-hospital mortality in AIS patients with atrial fibrillation, with an increased risk notably present at BUCR levels above 19.63 mg/dL[19]. Similarly, Liu et al. reported a U-shaped relationship in a Korean prospective cohort, where both low and high BUCR levels were associated with poor 3-month functional outcomes, and an inflection point was observed at 21.591 mg/dL[25]. These discrepancies may stem from both clinical context and methodological differences across studies. First, the clinical populations varied; for example, some studies exclusively included patients with atrial fibrillation, whereas others imposed no such restriction. Second, the definitions of outcomes differed—some studies assessed 3-month functional outcomes, whereas the present study focused on 28-day in-hospital mortality. Finally, variations in statistical modeling may also account for inconsistent findings. Specifically, the use of logistic regression versus Cox proportional hazards models and whether competing risk adjustment was applied could influence the observed associations between BUCR and clinical outcomes. Nevertheless, across diverse settings and study designs [19,25], the consistent association between elevated BUCR and adverse outcomes reinforces its potential as a clinically accessible and cost-effective prognostic marker. Our findings suggest that BUCR may function as a continuous indicator of systemic physiological stress rather than serving as a binary risk threshold.

The mechanisms underlying the associations between elevated BUCR and increased incidence and mortality of IS have not been fully elucidated. Nonetheless, several plausible pathophysiological pathways may account for this relationship. First, a high BUCR may reflect a catabolic state characterized by elevated BUN and reduced Scr, indicative of increased protein breakdown and decreased muscle mass [26,27]. These features are commonly observed in ICU or frail individuals experiencing metabolic stress [26,27]. Second, an elevated BUCR may also suggest impaired renal and cardiac perfusion [28,29], both of which are associated with adverse outcomes in stroke patients [30,31]. In our study, patients with higher BUCR levels presented elevated BUN and blood glucose, along with reduced creatinine, suggesting a state of catabolic stress and fluid imbalance. These abnormalities may act synergistically to increase short-term mortality risk in patients with IS. Third, elevated BUCR is widely recognized as a surrogate marker of dehydration, which increases blood viscosity and reduces cerebral perfusion [24,32]. However, our data show that patients with high BUCR have lower Scr levels, which contradicts the classic pattern of dehydration. Therefore, future research should explore potential confounding factors that may influence BUCR levels, such as renal function, medication use, and comorbidities, and further investigate the relationship between BUCR and stroke outcomes in larger, more representative populations.

This study is the first to reveal a significant positive association between BUCR and 28-day in-hospital mortality among ICU patients with IS via data from the eICU collaborative research database. As a large, multicenter, and high-quality dataset, the eICU enhances the generalizability and credibility of the findings. These results highlight the potential clinical utility of BUCR as a prognostic marker in IS. The associations remained consistent across clinical subgroups, with no significant interactions, supporting the robustness of the results. Furthermore, multiple sensitivity analyses—including complete-case analysis, MI, PSM, and E-value estimation—were conducted to ensure the reliability of the conclusions.

However, several limitations should be acknowledged. First, owing to its retrospective design, this study cannot establish a causal relationship between BUCR and patient outcomes. Second, the absence of critical prognostic factors, such as stroke severity, infarct size, thrombolysis status, stroke subtype, blood pressure, and liver disease, which were not available in the eICU database, represents a significant limitation of our study. These factors are well-established in influencing stroke outcomes, and their exclusion may impact the interpretation of our findings. Future studies that incorporate these variables would provide a more comprehensive evaluation of the prognostic value of BUCR in ischemic stroke patients. Third, this study could not adjust for potential confounders such as stroke severity (e.g., NIHSS), pre-stroke functional status, or etiology, which may influence the observed association between high BUCR and mortality. To evaluate the impact of unmeasured confounders, we conducted an E-value analysis, which indicated that a confounder would need a risk ratio of at least 2.61 with both BUCR and mortality to fully explain the observed association. Fourth, BUCR in our study was calculated based on a single measurement within 24 hours of ICU admission, without accounting for fluctuations in BUN and Scr levels due to resuscitation or early renal injury. These fluctuations may impact the accuracy of BUCR. Future studies could use more dynamic approaches to monitor renal function, offering a more accurate assessment of BUCR’s prognostic value. Fifth, the analysis was limited to 28-day mortality, without an evaluation of long-term outcomes or functional recovery. Finally, although eICU-CRD v2.0 represents a broad U.S. population, its lack of international data limits the external generalizability of the findings. Prospective, multicenter studies with stroke subtype classification and extended follow-up are needed to validate the prognostic value of BUCR in IS, including its role in predicting recurrence and guiding clinical risk stratification.

## Conclusion

An elevated BUCR is independently associated with increased short-term all-cause mortality in ICU patients with IS. These findings underscore the potential of BUCR as a simple, accessible, and clinically valuable biomarker for early risk stratification. Routine assessment and targeted management of BUN and Scr levels may aid in improving short-term outcomes in this high-risk population.

## Supporting information

S1 Table Distribution of variables with missing data. BMI,body mass index; DM, diabetes mellitus; SOFA, sequential organ failure assessment.
(DOCX)

S2 Table Assessment of collinearity among independent variables in the final regression model. BMI,body mass index; SOFA, sequential organ failure assessment; COPD, chronic obstructive pulmonary disease; CHF, congestive heart failure; AMI, acute myocardial infarction; DM, diabetes mellitus.
(DOCX)

S3 Table Basic characteristics of the population included in this study. BMI,body mass index; SOFA, sequential organ failure assessment; COPD, chronic obstructive pulmonary disease; CHF, congestive heart failure; AMI, acute myocardial infarction; DM, diabetes mellitus.
(DOCX)

S4 Table The univariate analysis of the baseline variables and 28-day hospital mortality. SOFA score, sequential organ failure assessment score; DM, diabetes mellitus; COPD, chronic obstructive pulmonary disease; CHF, congestive heart failure; AMI, acute myocardial infarction; BUN, Blood urea nitrogen; BUCR, blood urea nitrogen to creatinine ratio.
(DOCX)

S5 Table Cox regression models assessing the association between the BUCR and 28-day in-hospital mortality excluding missing data. Model 1: unadjusted; Model 2: adjusted for age, gender, and ethnicity; Model 3: adjusted for Model 2 plus, BMI, mechanical ventilation use, SOFA score, DM, sepsis, COPD, CHF, AMI, arrhythmia, pneumonia, serum potassium, and serum sodium levels.
(DOCX)

S6 Table Cox regression models for the association between the BUCR and 28-day in-hospital mortality using multiple imputation. Model 1: unadjusted; Model 2: adjusted for age, gender, and ethnicity; Model 3: adjusted for Model 2 plus, BMI, mechanical ventilation use, SOFA score, DM, sepsis, COPD, CHF, AMI, arrhythmia, pneumonia, serum potassium, and serum sodium levels.
(DOCX)

S1 Fig E-value plot assessing the risk ratios of the BUCR in relation to 28-day in-hospital mortality. Adjusted for age, gender, ethnicity, BMI, mechanical ventilation use, SOFA score, DM, sepsis, COPD, CHF, AMI, arrhythmia, pneumonia, serum potassium, and serum sodium levels. BMI,body mass index; SOFA, sequential organ failure assessment; COPD, chronic obstructive pulmonary disease; CHF, congestive heart failure; AMI, acute myocardial infarction; DM, diabetes mellitus.
(DOCX)
