# Supplementary material for: Elevated blood urea nitrogen-to-creatinine ratio predicts short-term mortality in intensive care unit patients with ischemic stroke: Evidence from a multicenter cohort
Source: PLoS One. 2025 Dec 4;20(12):e0337807. doi: 10.1371/journal.pone.0337807 (PMC12677572; doi:10.1371/journal.pone.0337807)
Supplement: S1 Table — BMI,body mass index; DM, diabetes mellitus; SOFA, sequential organ failure assessment. (DOCX) [file pone.0337807.s001.docx]

| **S1 Table. Distribution of variables with missing data.** | | |
| --- | --- | --- |
| **Variables** | **Number of Missi**ng | **Missing proportion(%)** |
| BMI | 110 | 3.40 |
| DM | 45 | 1.39 |
| Ethnicity | 173 | 5.35 |
| Serum potassium | 509 | 15.75 |
| Serum creatinine | 510 | 15.78 |
| Mechanical ventilation use | 45 | 1.39 |
| SOFA score | 2 | 0.06 |
| BMI,body mass index; DM, diabetes mellitus; SOFA, sequential organ failure assessment. | | |
