# Supplementary material for: Elevated blood urea nitrogen-to-creatinine ratio predicts short-term mortality in intensive care unit patients with ischemic stroke: Evidence from a multicenter cohort
Source: PLoS One. 2025 Dec 4;20(12):e0337807. doi: 10.1371/journal.pone.0337807 (PMC12677572; doi:10.1371/journal.pone.0337807)
Supplement: S2 Table — BMI,body mass index; SOFA, sequential organ failure assessment; COPD, chronic obstructive pulmonary disease; CHF, congestive heart failure; AMI, acute myocardial infarction; DM, diabetes mellitus. (DOCX) [file pone.0337807.s002.docx]

| **S2 Table. Assessment of collinearity among independent variables in the final regression model.** | | | | |
| --- | --- | --- | --- | --- |
| **Variables** | **GVIF** | **Df** | **Adjusted GVIF** | **Collinearity** |
| **Age** | 1.178 | 1 | 1.086 | No |
| **Gender** | 1.098 | 1 | 1.048 | No |
| **Ethnicity** | 1.088 | 2 | 1.021 | No |
| **BMI** | 1.069 | 1 | 1.034 | No |
| **Mechanical ventilation use** | 1.249 | 1 | 1.118 | No |
| **SOFA score** | 1.354 | 1 | 1.163 | No |
| **SEPSIS** | 1.108 | 1 | 1.052 | No |
| **COPD** | 1.074 | 1 | 1.036 | No |
| **CHF** | 1.107 | 1 | 1.052 | No |
| **AMI** | 1.026 | 1 | 1.013 | No |
| **DM** | 1.033 | 1 | 1.016 | No |
| **Arrhythmia** | 1.124 | 1 | 1.06 | No |
| **Pneumonia** | 1.089 | 1 | 1.044 | No |
| **Serum potassium** | 1.113 | 1 | 1.055 | No |
| **Serum sodium** | 1.162 | 1 | 1.078 | No |
| BMI,body mass index; SOFA, sequential organ failure assessment; COPD, chronic obstructive pulmonary disease; CHF, congestive heart failure; AMI, acute myocardial infarction; DM, diabetes mellitus. | | | | |
