# Supplementary material for: Elevated blood urea nitrogen-to-creatinine ratio predicts short-term mortality in intensive care unit patients with ischemic stroke: Evidence from a multicenter cohort
Source: PLoS One. 2025 Dec 4;20(12):e0337807. doi: 10.1371/journal.pone.0337807 (PMC12677572; doi:10.1371/journal.pone.0337807)
Supplement: S3 Table — BMI,body mass index; SOFA, sequential organ failure assessment; COPD, chronic obstructive pulmonary disease; CHF, congestive heart failure; AMI, acute myocardial infarction; DM, diabetes mellitus. (DOCX) [file pone.0337807.s003.docx]

| **S3 Table. Basic characteristics of the population included in this study.** | | | | |  |
| --- | --- | --- | --- | --- | --- |
| **Variables** | **Total  (n = 2,702)** | **28-day survivors  (n = 2,389)** | **28-day non-survivors  (n =313)** | **P value** |  |
| **Age, mean(SD), year** | 68.59 ± 14.29 | 68.34 ± 14.29 | 70.53 ± 14.13 | 0.011 |  |
| **Gender(male), n(%)** | 1293 (47.85) | 1145 (47.93) | 148 (47.28) | 0.830 |  |
| **Ethnicity, n(%)** |  |  |  | 0.445 |  |
| **Caucasian** | 2062 (80.99) | 1821 (80.86) | 241 (81.97) |  |  |
| **African American** | 293 (11.51) | 265 (11.77) | 28 (9.52) |  |  |
| **Other or unknown** | 191 (7.50) | 166 (7.37) | 25 (8.5) |  |  |
| **BMI, mean(SD), kg/m^2^** | 28.72 ± 7.19 | 28.69 ± 7.19 | 28.93 ± 7.17 | 0.581 |  |
| **Mechanical ventilation use, n(%)** | 558 (20.94) | 396 (16.82) | 162 (52.09) | < 0.001 |  |
| **SOFA score** | 2.00  (1.00, 4.00) | 2.00  (1.00, 3.00) | 4.00  (3.00, 6.00) | < 0.001 |  |
| **SEPSIS, n(%)** | 76 (2.81) | 54 (2.26) | 22 (7.03) | < 0.001 |  |
| **COPD, n(%)** | 127 (4.70) | 102 (4.27) | 25 (7.99) | 0.003 |  |
| **CHF, n(%)** | 147 (5.44) | 120 (5.02) | 27 (8.63) | 0.008 |  |
| **AMI, n(%)** | 82 (3.03) | 64 (2.68) | 18 (5.75) | 0.003 |  |
| **Diabetes mellitus, n(%)** | 559 (20.98) | 490 (20.82) | 69 (22.19) | 0.577 |  |
| **Pneumonia, n(%)** | 210 (7.77) | 155 (6.49) | 55 (17.57) | < 0.001 |  |
| **Arrhythmia, n (%)** | 548 (20.28) | 448 (18.75) | 100 (31.95) | < 0.001 |  |
| **Glucose, mean(SD), mg/dL** | 138.52 ± 65.21 | 134.72 ± 61.50 | 167.65 ± 83.18 | < 0.001 |  |
| **BUN, median(IQR), mg/dL** | 17.00 (12.00, 24.00) | 16.00  (12.00, 23.00) | 22.00  (16.00, 31.00) | < 0.001 |  |
| **Serum creatinine, median(IQR), mg/dl** | 0.91  (0.74, 1.21) | 0.90  (0.72, 1.20) | 1.10  (0.83, 1.50) | < 0.001 |  |
| **Serum potassium, mean(SD), mmol/L** | 3.97 ± 0.57 | 3.96 ± 0.54 | 4.05 ± 0.71 | 0.009 | |
| **Serum sodium, mean(SD), mmol/L** | 139.07 ± 4.11 | 139.02 ± 3.99 | 139.44 ± 4.91 | 0.092 | |
| **BUCR** | 19.04 ± 8.49 | 18.76 ± 8.22 | 21.15 ± 10.14 | < 0.001 | |
| BMI,body mass index; SOFA, sequential organ failure assessment; COPD, chronic obstructive pulmonary disease; CHF, congestive heart failure; AMI, acute myocardial infarction; DM, diabetes mellitus; | | | | | |
