# Supplementary material for: Elevated blood urea nitrogen-to-creatinine ratio predicts short-term mortality in intensive care unit patients with ischemic stroke: Evidence from a multicenter cohort
Source: PLoS One. 2025 Dec 4;20(12):e0337807. doi: 10.1371/journal.pone.0337807 (PMC12677572; doi:10.1371/journal.pone.0337807)
Supplement: S4 Table — SOFA score, sequential organ failure assessment score; DM, diabetes mellitus; COPD, chronic obstructive pulmonary disease; CHF, congestive heart failure; AMI, acute myocardial infarction; BUN, Blood urea nitrogen; BUCR, blood urea nitrogen to creatinine ratio. (DOCX) [file pone.0337807.s004.docx]

| **S4 Table. The univariate analysis of the baseline variables and 28-day hospital mortality.** | | |
| --- | --- | --- |
| **Variables** | **Hazard ratios (95% CI)** | **P-value** |
| **Age (years)** |  |  |
| <60 | 1 |  |
| >=60 | 1.018 (1.009, 1.026) | < 0.001 |
| **Gender** |  |  |
| Male | 1 |  |
| Female | 0.929 (0.754, 1.146) | 0.492 |
| **Ethnicity** |  |  |
| Caucasian | 1 |  |
| African American | 0.597 (0.409, 0.871) | 0.007 |
| Others/Unkown | 0.917 (0.622, 1.354) | 0.664 |
| **BMI** | 1.001 (0.9865, 1.0153) | 0.915 |
| **Mechanical ventilation use** |  |  |
| No | 1 |  |
| Yes | 2.439 (1.969, 3.021) | < 0.001 |
| **SOFA score** | 1.209 (1.167, 1.252) | < 0.001 |
| **DM** |  |  |
| No | 1 |  |
| Yes | 0.98 (0.763, 1.26) | 0.876 |
| **COPD** |  |  |
| No | 1 |  |
| Yes | 1.369 (0.924, 2.028) | 0.117 |
| **CHF** |  |  |
| No | 1 |  |
| Yes | 1.316 (0.91, 1.904) | 0.145 |
| **AMI** |  |  |
| No | 1 |  |
| Yes | 1.326 (0.824, 2.133) | 0.245 |
| **Pneumonia** |  |  |
| No | 1 |  |
| Yes | 1.304 (0.974, 1.746) | 0.075 |
| **Arrhythmia** |  |  |
| No | 1 |  |
| Yes | 1.322 (1.05, 1.663) | 0.017 |
| **Serum creatinine (mg/dL) Tertile** |  |  |
| 0.24－0.9 | 1 |  |
| 0.91－16.1 | 1.036 (0.969, 1.107) | 0.297 |
| **BUN (mg/dL) Tertile** |  |  |
| 4－16 | 1 |  |
| 17－144 | 1.009 (1.004, 1.015) | < 0.001 |
| **Serum potassium (mmol/L) Tertile** |  |  |
| 2.4－3.9 | 1 |  |
| 4.0－7.4 | 1.147 (0.92, 1.43) | 0.224 |
| **Serum sodium (mmol/L) Tertile** |  |  |
| 116－139 | 1 |  |
| 140－160 | 0.991 (0.794, 1.237) | 0.937 |
| **BUCR Tertile** |  |  |
| Q1 | 1 |  |
| Q2 | 1.402 (1.041, 1.89) | 0.026 |
| Q3 | 1.714 (1.298, 2.263) | < 0.001 |
| SOFA score, sequential organ failure assessment score; DM, diabetes mellitus; COPD, chronic obstructive pulmonary disease; CHF, congestive heart failure; AMI, acute myocardial infarction; BUN, Blood urea nitrogen; BUCR, blood urea nitrogen to creatinine ratio. | | |
