# Supplementary material for: Elevated blood urea nitrogen-to-creatinine ratio predicts short-term mortality in intensive care unit patients with ischemic stroke: Evidence from a multicenter cohort
Source: PLoS One. 2025 Dec 4;20(12):e0337807. doi: 10.1371/journal.pone.0337807 (PMC12677572; doi:10.1371/journal.pone.0337807)
Supplement: S5 Table — Model 1: unadjusted; Model 2: adjusted for age, gender, and ethnicity; Model 3: adjusted for Model 2 plus, BMI, mechanical ventilation use, SOFA score, DM, sepsis, COPD, CHF, AMI, arrhythmia, pneumonia, serum potassium, and serum sodium levels. (DOCX) [file pone.0337807.s005.docx]

| **S5 Table. Cox regression models assessing the association between the BUCR and 28-day in-hospital mortality excluding missing data.** | | | | | | | |
| --- | --- | --- | --- | --- | --- | --- | --- |
| **Variables** | **Event, (n%)** | **Model1** | | **Model2** | | **Model3** | |
|  |  | **HR (95% CI)** | **P value** | **HR (95% CI)** | **P value** | **HR (95% CI)** | **P value** |
| BUCR index | | | | | | | |
| Continuous | 283 (11.6) | 1.017  (1.006, 1.028) | 0.002 | 1.013  (1.001, 1.025) | 0.033 | 1.014  (1.002, 1.026) | 0.021 |
| Quartile |  |  |  |  |  |  |  |
| Q1 | 69 (8.6) | 1(Ref) |  | 1(Ref) |  | 1(Ref) |  |
| Q2 | 90 (11.1) | 1.495  (1.092, 2.046) | 0.012 | 1.323  (0.959, 1.826) | 0.088 | 1.586  (1.14, 2.205) | 0.006 |
| Q3 | 124 (15.3) | 1.775  (1.322, 2.383) | < 0.001 | 1.511  (1.107, 2.063) | 0.009 | 1.616  (1.173, 2.226) | 0.003 |
| P for trend |  | 1.318  (1.143, 1.52) | < 0.001 | 1.22  (1.048, 1.42) | 0.010 | 1.244  (1.067, 1.451) | 0.005 |

Model 1: unadjusted

Model 2: adjusted for age, gender, and ethnicity

Model 3: adjusted for Model 2 plus, BMI, mechanical ventilation use, SOFA score, DM, sepsis, COPD, CHF, AMI, arrhythmia, pneumonia, serum potassium, and serum sodium levels.
