# Supplementary material for: Elevated blood urea nitrogen-to-creatinine ratio predicts short-term mortality in intensive care unit patients with ischemic stroke: Evidence from a multicenter cohort
Source: PLoS One. 2025 Dec 4;20(12):e0337807. doi: 10.1371/journal.pone.0337807 (PMC12677572; doi:10.1371/journal.pone.0337807)
Supplement: S6 Table — Model 1: unadjusted; Model 2: adjusted for age, gender, and ethnicity; Model 3: adjusted for Model 2 plus, BMI, mechanical ventilation use, SOFA score, DM, sepsis, COPD, CHF, AMI, arrhythmia, pneumonia, serum potassium, and serum sodium levels. (DOCX) [file pone.0337807.s006.docx]

| **S6 Table. Cox regression models for the association between the BUCR and 28-day in-hospital mortality using multiple imputation.** | | | | | | | |
| --- | --- | --- | --- | --- | --- | --- | --- |
| **Variables** | **Event, (n%)** | **Model1** | | **Model2** | | **Model3** | |
|  |  | **HR (95% CI)** | **P value** | **HR (95% CI)** | **P value** | **HR (95% CI)** | **P value** |
| BUCR index | | | | | | | |
| Continuous | 353 (10.9) | 1.019  (1.01, 1.029) | <0.001 | 1.015  (1.005, 1.026) | 0.005 | 1.016  (1.006, 1.027) | 0.003 |
| Quartile |  |  |  |  |  |  |  |
| Q1 | 88 (8.2) | 1(Ref) |  | 1(Ref) |  | 1(Ref) |  |
| Q2 | 108 (10) | 1.384  (1.044, 1.835) | 0.024 | 1.238  (0.929, 1.65) | 0.145 | 1.466  (1.094, 1.965) | 0.010 |
| Q3 | 157 (14.6) | 1.754  (1.351, 2.278) | <0.001 | 1.5  (1.139, 1.976) | 0.004 | 1.584  (1.194, 2.101) | 0.001 |
| P for trend |  | 1.319  (1.161, 1.499) | <0.001 | 1.224  (1.068, 1.402) | 0.004 | 1.243  (1.084, 1.425) | 0.002 |

Model 1: unadjusted

Model 2: adjusted for age, gender, and ethnicity

Model 3: adjusted for Model 2 plus, BMI, mechanical ventilation use, SOFA score, DM, sepsis, COPD, CHF, AMI, arrhythmia, pneumonia, serum potassium, and serum sodium levels.
