# Supplementary material for: Elevated blood urea nitrogen-to-creatinine ratio predicts short-term mortality in intensive care unit patients with ischemic stroke: Evidence from a multicenter cohort
Source: PLoS One. 2025 Dec 4;20(12):e0337807. doi: 10.1371/journal.pone.0337807 (PMC12677572; doi:10.1371/journal.pone.0337807)
Supplement: S1 Fig — Adjusted for age, gender, ethnicity, BMI, mechanical ventilation use, SOFA score, DM, sepsis, COPD, CHF, AMI, arrhythmia, pneumonia, serum potassium, and serum sodium levels. BMI,body mass index; SOFA, sequential organ failure assessment; COPD, chronic obstructive pulmonary disease; CHF, congestive heart failure; AMI, acute myocardial infarction; DM, diabetes mellitus. (DOCX) [file pone.0337807.s007.docx]

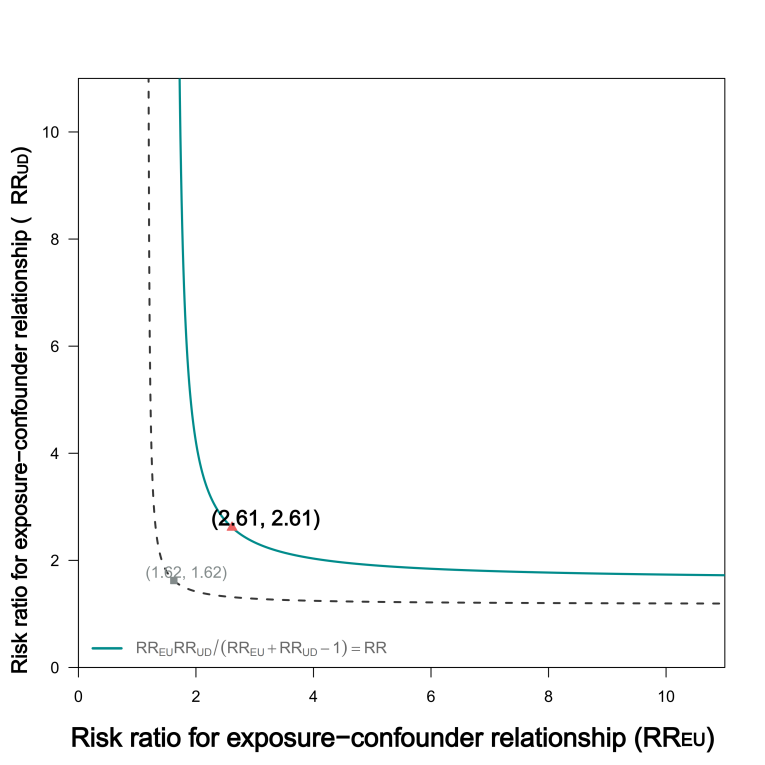


**S1 Fig. E-value plot assessing the risk ratios of the BUCR in relation to 28-day in-hospital mortality.** Adjusted for age, gender, ethnicity, BMI, mechanical ventilation use, SOFA score, DM, sepsis, COPD, CHF, AMI, arrhythmia, pneumonia, serum potassium, and serum sodium levels. BMI,body mass index; SOFA, sequential organ failure assessment; COPD, chronic obstructive pulmonary disease; CHF, congestive heart failure; AMI, acute myocardial infarction; DM, diabetes mellitus.
